# Impact of Medical Doctors Global Health and Tropical Medicine on decision-making in caesarean section: a pre- and post-implementation study in a rural hospital in Malawi

**DOI:** 10.1186/s12960-020-00516-5

**Published:** 2020-11-09

**Authors:** Wouter Bakker, Emma Bakker, Christiaan Huigens, Emily Kaunda, Timothy Phiri, Jogchum Beltman, Jos van Roosmalen, Thomas van den Akker

**Affiliations:** 1 Clinical Department, St. Luke’s Hospital, Malosa, Malawi; 2 Athena Institute, VU University Amsterdam, Amsterdam, The Netherlands; 3 Department of Obstetrics and Gynaecology, Leiden University Medical Centre, Leiden, The Netherlands; 4 Nursing and Midwifery Department, St. Luke’s Hospital, Malosa, Malawi

**Keywords:** Caesarean section, Audit, MDGHTM, Associate clinicians, Unnecessary caesareans

## Abstract

**Background:**

Medical doctors with postgraduate training in Global Health and Tropical Medicine (MDGHTM) from the Netherlands, a high-income country with a relatively low caesarean section rate, assist associate clinicians in low-income countries regarding decision-making during labour. Objective of this study was to assess impact of the presence of MDGHTMs in a rural Malawian hospital on caesarean section rate and indications.

**Methods:**

This retrospective pre- and post-implementation study was conducted in a rural hospital in Malawi, where MDGHTMs were employed from April 2015. Indications for caesarean section were audited against national protocols and defined as supported or unsupported by these protocols. Caesarean section rates and numbers of unsupported indications for the years 2015 and 2016 per quarter for different staff cadres were assessed by linear regression.

**Results:**

Six hundred forty-five women gave birth by caesarean section in the study period. The caesarean rate dropped from 20.1 to 12.8% (*p* < 0.05, *R*^2^ = 0.53, *y* = − 0.0086x + 0.2295). Overall 132 of 501 (26.3%) auditable indications were not supported by documentation in medical records. The proportion of unsupported indications dropped significantly over time from 47.0 to 4.4% (*p* < 0.01, *R*^2^ = 0.71, *y* = − 0.0481x + 0.4759). Stratified analysis for associate clinicians only (excluding caesarean sections performed by medical doctors) showed a similar decrease from 48.3 to 6.5% (*p* < 0.05, *R*^2^ = 0.55, *y* = − 0.0442x + 0.4805).

**Conclusions:**

Our results indicate that presence of MDGHTMs was accompanied by considerable decreases in caesarean section rate and proportion of unsupported indications for caesarean section in this facility. Their presence is likely to have influenced decision-making by associate clinicians.

## Background

The proportion of caesarean births is increasing worldwide, in both high- and low-income settings [1–3]. A worldwide ecological study representing 98% of global live births found no important association with maternal and neonatal mortality at caesarean section rates higher than 10%, in line with a systematic review by the World Health Organization and their statement on caesarean sections [4–6]. Caesarean sections may be associated with severe maternal outcomes like massive blood transfusion, admission to intensive care unit, hysterectomy, and death, and these associations are strongest in sub-Saharan Africa [7–9]. The risk of uterine rupture in subsequent pregnancies is increased, which is particularly problematic in countries with high fertility rates such as Malawi [10–14]. Therefore, it remains crucial to carefully select those women eligible for surgical intervention and prevent caesarean sections without medical indication, for instance by following evidence-based indications and performing audit of indications [2]. In addition, the World Health Organization advises to monitor caesarean section rates at a facility level [6].

In the Medical Doctor Global Health and Tropical Medicine (MDGHTM) programme in the Netherlands, participants are trained to work as medical doctors in low-resource settings [15, 16]. Their postgraduate training has a duration of approximately 3 years and focuses on acquiring basic surgical and obstetric skills, as well as knowledge of tropical diseases and public health [17, 18]. MDGHTMs are expected to critically assess labour progress and indications for obstetric interventions, and are prepared to provide clinical assistance and onsite training to other (associate) clinicians in hospitals in low-income countries. MDGHTMs often work in under-staffed rural areas in low-resource settings and combine clinical, managerial, and educational tasks [16, 19]. However, the impact of their presence on obstetric management in their work environment has never been systematically assessed.

The first objective of this study was to describe the caesarean section rate during the period of introduction of MDGHTMs in a rural hospital in Malawi, where hospital management was concerned about increased caesarean section rates. Second objective was to examine whether there was a decrease in the proportion of caesarean sections that were not supported by medical indications.

## Methods

### Study site

This pre- and post-implementation study was conducted at St. Luke’s Hospital in Malosa. This 150-bed rural hospital is based in the southern region of Malawi, a landlocked low-resource country with a population over 17 million. St. Luke’s Hospital is a mission facility run by the Christian Health Association of Malawi and works with a principle of user fees for services, but maternity care is free of charge for the catchment population through a government sponsorship programme. The hospital serves a catchment population of roughly 30 000 and offers comprehensive emergency obstetric care, with an average number of 2000 births per year. The monthly institutional caesarean section rate varied between 20 and 30%, which alerted hospital management and led to plans for further investigation [20, 21]. Services are usually provided by associate clinicians, in Malawi called ‘clinical officers’. Clinical officers followed a 4-year practice-oriented training in general medicine and basic surgical procedures [22, 23]. The hospital struggled to contract a medical doctor, until April 2015, when an MDGHTM was employed in the facility, followed by a second MDGHTM from the beginning of 2016 onwards.

**Fig. 1:**
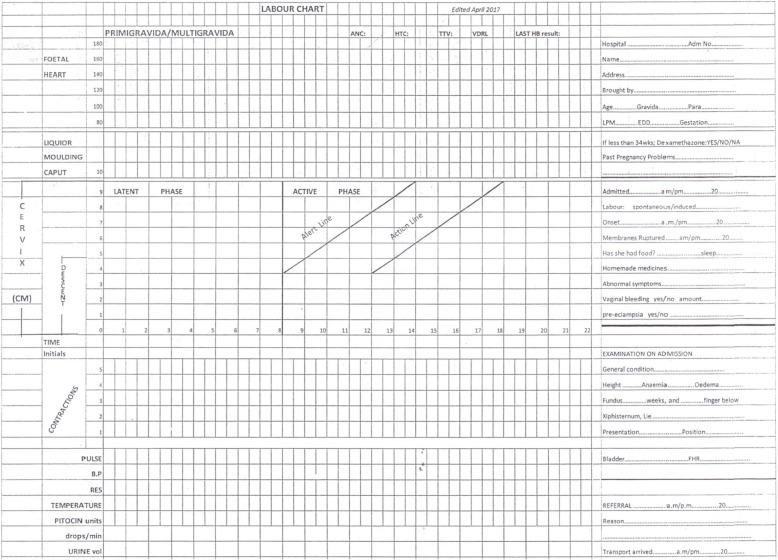
The labour chart with partograph used in St Luke’s Hospital, Malawi, adapted from the modified WHO partograph

The labour ward of St. Luke’s hospital has five beds with an average of two midwives per shift. Foetal monitoring is done by intermittent auscultation using Pinard’s fetoscope or Doppler. Routinely, vaginal examination for progress is performed 4-hourly. Women are admitted in the labour ward when in active labour, usually from a dilatation of the cervix of at least 4 cm. Progress and foetal heart rates are plotted in a partograph, as enforced by the Ministry of Health (Fig. 1: partograph used in Malawi). Cardiotocography was not available.

### MDGHTM

Since the late 1960s, the Netherlands Society for Tropical Medicine and International Health offers a training programme for doctors who are planning to work in low-resource settings. The programme, originally consisting of extra training in surgery and obstetrics followed by a 3-month course in Tropical Medicine and Hygiene on top of basic medical training, aimed to equip medical doctors with some extra skills much needed in low-resource settings where an all-round approach is required. The programme evolved into formal specialty training for MDGHTM, which officially started on January 1, 2014. The programme has now two possible pathways: 9 months of obstetrics/gynaecology combined with either 9 months of surgery or paediatrics, followed by the Tropical Medicine and Hygiene course and a 6-month placement in a low-resource setting.

St. Luke’s hospital has a longstanding history with employing Dutch doctors. After a short period without any medical doctor present in the hospital, the hospital started employing MDGHTMs again from mid-2015. Next to clinical tasks, they take part in management as senior medical officer and fulfil a supportive and supervising role for junior staff. These doctors work alongside the clinical officers, but also have a leadership responsibility as head of the team.

### Data collection

Medical records were collected of all women who gave birth between January 1, 2015, and December 31, 2016. These records consisted of partographs and information on admission and follow-up. The partograph is a management tool for monitoring labour, containing all patient characteristics and keeping track of labour progress (Fig. 1). Women were included based on the presence of the partograph in their records. All basic characteristics were collected, including indication, type of health worker who made the decision for caesarean section, progress of labour, timing of the procedure, pre-operative interventions as artificial rupture of membranes, augmentation with oxytocin and trial of instrumental vaginal birth in the second stage of labour, and maternal and perinatal complications. Unless clearly stated otherwise, the health worker who was listed as the surgeon in the procedure was labelled as the one who took the decision. This is according to hospital routine. Files were analysed by one of the researchers present in the workplace, but if information was unclear, cases were discussed with one of the other research team members (WB or ACH).

### Comparison to protocols

Staff of St Luke’s hospital follow the National Obstetrics & Gynaecology Protocols and Guidelines, which include indications for caesarean section. For all of these indications, literature study and consultation of local clinicians was performed in order to provide measurable criteria (Table 1). Every caesarean section with an indication categorised in Table 1 was audited using these criteria. Cases in which criteria for any indication were not met, were labelled as unsupported indications. Cases in which it was impossible to assess whether indication met criteria due to missing data, were labelled ‘unable to assess’. This was, for example, the case when the indication was foetal distress in absence of foetal heart rate recordings or if there was a delay between the last documentation and decision of caesarean section of more than 8 h. Cases that had indications not listed in Table 1 (the indications in this table are labelled ‘auditable indications’) were collected in the database but not audited. No distinction was made between elective and emergency caesarean section, since no system of booking caesarean sections was in place in the study period.

**Table 1:** Auditable indications based on national protocols

Indication	Criteria
Indications related to prolonged labour *For example:* *Obstructed labour (as an extreme form of prolonged labour)* *Cephalopelvic disproportion* *Prolonged first stage of labour* *Prolonged second stage of labour* *Cervical dystocia* *Oedematous cervix*	Prolonged 1st stage = non-progressing dilatation > 2 h in case of ruptured membranes + at least three moderate contractions/10 min. Prolonged 2nd stage = duration second stage > 1 h
Foetal distress	Foetal heart rate < 110 or > 170 beats per minute for > 1 min on intermittent auscultation, in between contractions.
Foetal malpresentation	Perioperative foetal presentation = Breech Brow presentation Face presentation (mento-posterior) Compound presentation Transverse
Two or more previous scars	Two or more previous caesarean sections documented in history in partograph

### Outcome

Primary outcome measure was quarterly caesarean section rate in the period under review. Caesarean section rate was defined as the number of caesarean sections divided by the total number of women who gave birth. Secondary outcome measures were frequencies of different indications for caesarean sections, percentage of unsupported indications over time, percentage of unsupported indications by clinical officers, and maternal and perinatal complication rates per period.

### Data analysis

The database was checked for errors and duplicates. Data analysis was performed using IBM SPSS Statistics Version 24 and Microsoft Excel Version 16.24. Total number (*n*) and frequency (%) of demographic variables and clinical characteristics were analysed. Caesarean section rates, number of unsupported indications, and maternal and neonatal complication rates were calculated for the eight quarters of the study period.

## Results

**Fig. 2:**
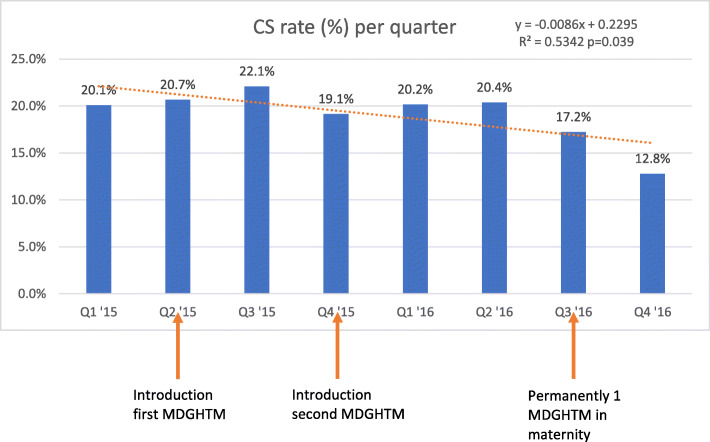
Caesarean section rate per quarter

Medical records of 3428 births were retrieved, of which 645 were caesarean sections (caesarean section rate 18.8%). Table 2 shows clinical characteristics of women who underwent caesarean section. Figure 2 shows a temporal trend of a reduced caesarean section rate per quarter during the study period (*y* = 0.0086x + 0.2295, *p* = 0.039). Medical officers performed 82 caesareans, clinical officers 513.

**Table 2:** Basic characteristics

Characteristics	*N*	%	Characteristics	*N*	%
**Age (years)**			**Gestational age (weeks)**		
< 20	169	26.2	< 32	10	1.6
20–24	184	28.5	32–34	20	3.1
25–29	124	19.2	35–36	110	17.1
30–34	81	12.6	37–39	183	28.4
≥ 35	63	9.8	≥ 40	33	5.1
Unknown	24	3.7	Unknown	289	44.8
Total	645	100	Total	645	100
Mean	24.4 (SD 6.34)		Mean	37.0 (SD 2.30)	
**Parity**			**Previous CS**		
0	253	39.2	0	466	72.2
1–2	251	38.9	1	120	18.6
≥ 3	123	19.1	≥ 2	42	6.5
Unknown	18	2.8	Unknown	17	2.6
Total	645	100	Total	645	100
Mean	1.37 (SD 1.63)		Mean	0.33 (SD 0.61)	
**Birthweight (g)**			**Type of clinician**		
< 1 500	10	1.5	Medical officer	82	12.7
1 500–1 999	25	3.7	Clinical officer	518	80.3
2 000–2 499	69	10.1	Unknown	45	7.0
2 500–2 999	174	25.5	Total	645	100
3 000–3 499	238	34.9	**Timing of CS**		
≥ 3 500	110	16.1	Office hours (08.00–17.00)	323	50.1
Unknown	56	8.2	Outside office hours	309	47.9
Total	682 (37 twin gestations)	100	Unknown	13	2.0
Mean	2944 g (SD 563 g)		Total	645	100

*CS* caesarean section

The commonest indication for caesarean section was cephalopelvic disproportion (CPD; 23.5%), followed by foetal distress (12.7%) and prolonged first stage of labour (of unknown reason; 12.2%). Nine percent of caesarean sections were performed because of two previous caesarean scars. All indications are listed in Table 3.

**Table 3:** Indications for caesarean section as described in case file

Indication	*N*	%
Cephalopelvic disproportion	150	23.3
Foetal distress	82	12.7
Prolonged first stage of labour	79	12.2
Two or more previous CS	57	8.8
Foetal malpresentation	53	8.2
Prolonged second stage of labour	30	4.7
Failed VBAC	20	3.1
Obstructed labour	16	2.5
Eclampsia	14	2.2
APH	12	1.9
Pre-eclampsia	10	1.6
Cervical dystocia	10	1.6
Cord prolapse	9	1.4
Abruptio placentae	7	1.1
Other	42	7.0
Missing	54	8.4
Total	645	100.0

Other indications were as follows: Retained second twin, premature rupture of membranes in HIV, extensive vulvovaginal warts, bad obstetric history, intra-uterine growth restriction, twin gestation in primigravida, placenta praevia, fresh caesarean scar, oligohydramnios, failed induction of labour, big fundus, previous myomectomy, active herpes infection, anhydramnios, chorioamnionitis, postdate pregnancy, epileptic convulsions, intra-uterine death with bilateral tubal ligation, stillbirth in twin gestation, cervical oedema

*CS* caesarean section

**Fig. 3:**
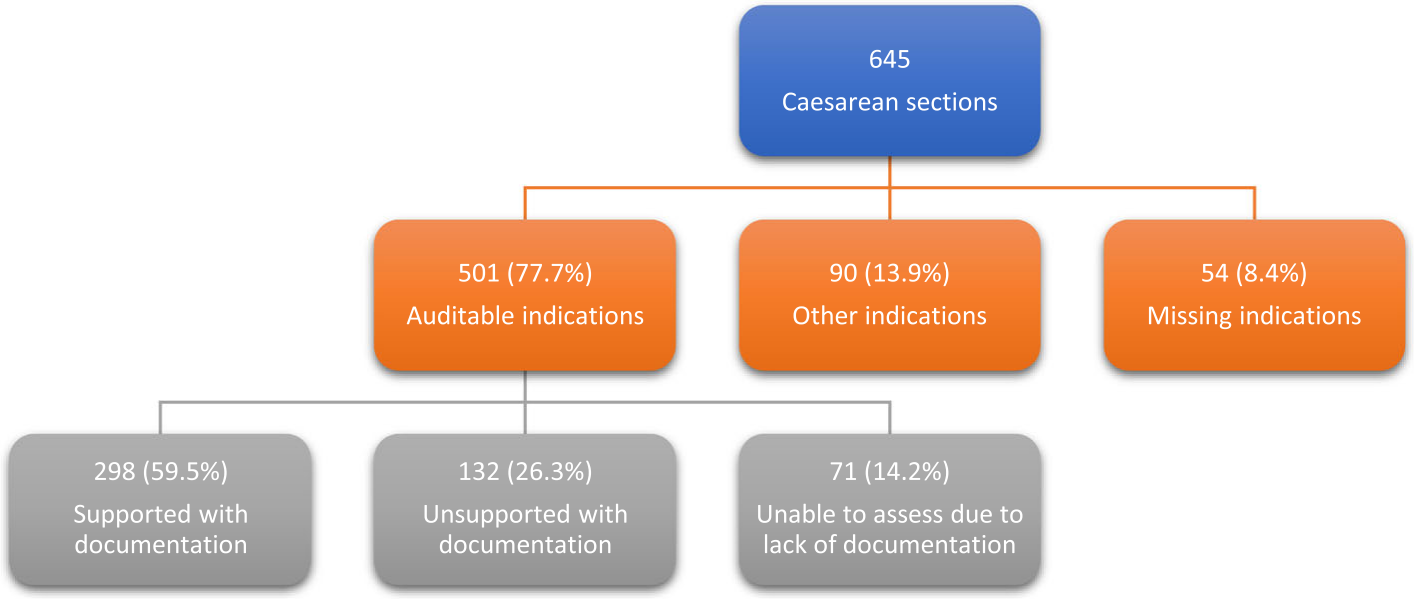
Distribution of caesarean section cases

Of 645 caesarean sections, 501 had an auditable indication: 90 (13.9%) had indications that were not auditable (not part of the criteria listed in table 1) and 54 (8.4%) did not have a documented indication (Table 3). One hundred and thirty-two (26.3%) of the audited cases had an unsupported indication: documentation in partograph and file did not meet criteria for that specific indication. In 71 (14.2%), too little information could be found in the partograph to assess the indication properly. In 298 (59.5%) of the audited cases, partograph and file provided evidence to justify the indication (see Fig. 3).

**Fig. 4:**
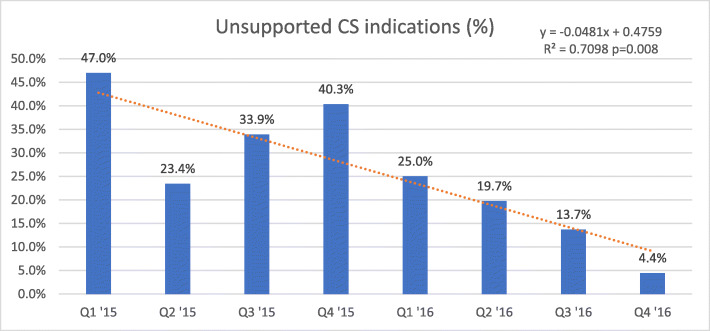
Unsupported indications per period

**Fig. 5:**
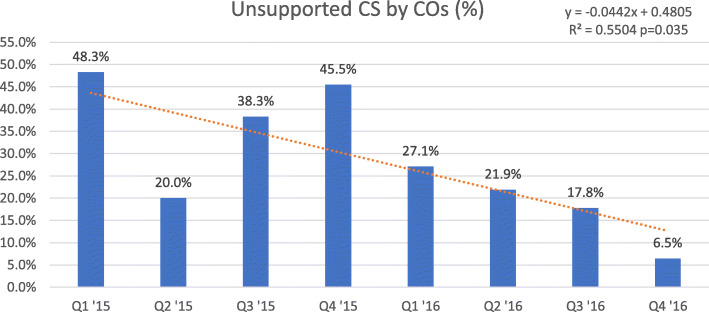
Proportion of unsupported caesarean sections by clinical officers

Fig. 4 shows the proportion of unsupported indications among the total auditable caesarean sections. There was a significant downward trend over time (*y* = − 0.0481 + 0.4759, *p* = 0.008). This trend was not only seen in all caesarean sections, but also in those performed by clinical officers (Fig. 5).

Looking into the different groups of indications, 93 (70.5%) of the caesarean sections with unsupported indications were due to prolonged labour. In these women, the partograph did not show signs of prolonged labour, although caesarean section was performed for that indication. Seventeen (12.9%) of the unsupported indications were foetal distress, 11 (8.3%) foetal malpresentation, and 11 (8.3%) two previous scars.

**Fig. 6:**
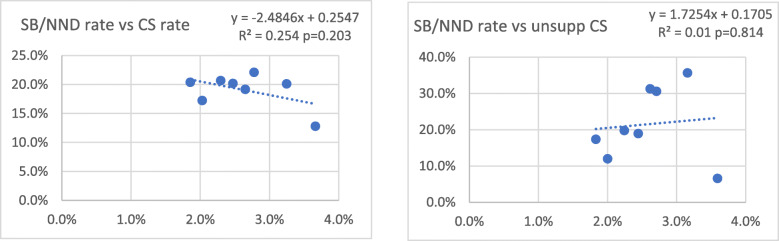
Combined stillbirth/neonatal death rate vs caesarean section rate and unsupported caesarean section rate

Maternal and perinatal outcome before moment of discharge, usually 3 days postpartum, remained relatively stable over time, although varying slightly per quarter. In the study period, four maternal deaths (0.6%), 15 fresh stillbirths (2.3%), five macerated stillbirths (0.8%), and six early neonatal deaths (0.9%) occurred in women undergoing caesarean section. Supplement 1 shows maternal and perinatal outcomes of caesarean section compared to vaginal birth. Stillbirth and neonatal death rates did not change significantly over time (*p* = 0.455 and 0.292 respectively, see Supplement 1). Plotting stillbirth and early neonatal death rates with caesarean section percentages (Fig. 6), no significant trend could be identified (*p* = 0.203) suggesting the decreasing caesarean section rate had no impact on these rates.

## Discussion

Our data suggest that the introduction of an MDGHTM could have had an effect on the caesarean section rate in the facility and on the proportion of caesarean sections not supported by documentation in medical records. There was a declining trend in both these rates over the 2-year study period. Introduction of an MDGHTM took place in the first quarter of 2015, and from the first quarter of 2016, there were two MDGHTMs working regularly in maternity care in the hospital. Additionally, from halfway the third quarter of 2016, there was one MDGHTM covering the maternity department daily. No other major changes occurred in the study period. Both caesarean section rate and proportion of unsupported indications decreased gradually. While medical officers took up some of the caesarean sections and thereby might have had an influence on the indications, remarkably the number of unsupported indications by associate clinicians decreased significantly as well. This is most likely attributed to supervision, training, and audit taking place throughout the study period, which were all part of the job description of the MDGHTMs. As part of hospital management, they fulfil a role as supervisor and head of the clinical team. In morning handovers, caesarean section indications were discussed and labour management was also part of maternal and perinatal morbidity and mortality audits. The effect on the overall caesarean rate could be partly attributed to this leadership role, but since the rate mostly decreased in the last two quarters, it could also be contributed to having an MDGHTM continuously in the maternity department to aid and supervise the midwifery team and thereby having a significant role in preventing unnecessary caesarean sections. Which element contributed most to the decline warrants further assessment.

A significant number of auditable cases (71, 14.3%) could not be audited due to lack of information. Indications for these cases might as well be considered unsupported, since no supporting information was found. Additionally, in 8.4% of all cases, caesarean section was performed with unknown indication. Therefore, our numbers of unsupported indication could be underestimated and stress the importance of evidence-based clinical decision-making to an even greater extent. Also, some criteria in national protocols give room to perform caesarean section at a rather low threshold. For example, a one-time foetal tachycardia can be interpreted as an opportunity to decide for caesarean section.

Reducing the number of unsupported, and possibly unnecessary, caesarean sections is important. It may save women from unnecessary risks of abdominal surgery. Furthermore, considering that a great number of procedures was done on young nulliparous women, preventing unnecessary procedures could prevent serious complications in subsequent pregnancies, especially considering the high fertility rate in Malawi. It seems a role exists for MDGHTMs, together with associate clinicians, to discuss indications for caesarean section and work towards optimal case selection. While this is a single-centre study, results are comparable with other caesarean section audits in similar settings, for example in Tanzania [24, 25]. However, the effect of MDGHTM was not studied previously. More research could provide better insights in this role, for example a qualitative study of health workers’ perceptions in settings where MDGHTMs are employed, preferably accompanied by quantitative and qualitative data on decision-making in caesarean sections.

A surprising result was the high number of caesarean sections performed in preterm labour, especially in the range of 35 to 37 weeks (17.1%). Since there is no policy for determination of gestational age by ultrasound in Malawi, the first day of the last menstrual period is used to calculate gestational age. This might not always be reliable and can for example be influenced by depot contraceptive usage or long-term breastfeeding after previous pregnancies. In a significant majority, gestational age was completely unknown, but the majority of birthweights (76.5%) was above 2500 g, suggesting that the number of very preterm births by caesarean is limited.

A major limitation of the study was lack of documentation. Inclusion was done based on complete partographs. While case notes with complete unfilled partographs were not included, it sometimes still remains unclear whether a partograph contained all information supporting the decision for caesarean section. For example, a last foetal heart rate or vaginal examination prior to the procedure on which the decision of foetal distress was made, may have been forgotten to be documented, leading to cases being labelled as unknown. Furthermore, there may be underreporting of cases due to missing case files or partographs, which we believe would be on a random basis, but still may influence the calculated caesarean section rates. Poor documentation during labour not only provides a challenge for data analysis, it also burdens the clinician making the decision for caesarean section. If little is known about progress or foetal condition, one may decide more quickly to proceed to caesarean delivery. Proper and regular documentation assists clinicians in making correct decisions and thereby also attributes to prevent unnecessary caesareans. This is also the case with documentation of complications, which seem quite low based on our collected data. Complications can be missed due to lack of a structured patient file administration, whereby readmissions were not connected to previous admissions.

We cannot be certain that other variables in the hospital stayed the same during the study period, although our analysis was based on that assumption. Staff or policy changes may influence the decision-making process. Over the study period, there were, however, no major changes in management, protocols, or resource availability. Since the study setting is a single rural facility, small numbers or individuals could have influence on certain changes over time. The increase in caesarean section rates seen in the second and third quarters of 2015 remains unexplained, but might be influenced by staff changes. It is therefore also challenging to predict the sustainability of the observed effect. Supervision and training may have long-term effects, especially with the enrolment of surgical and obstetric training for associate clinicians [22, 23, 26]. Effectivity of task-sharing with associate clinicians has been studied previously, but not on the matter of indications and caesarean section rate [22, 27].

Ideally, interventions require to have a sustainable impact on quality of care. While most likely the tradition of Dutch MDGHTM in St. Luke’s Hospital will continue for the foreseeable future, we hope that there is also a sustainable effect on clinical officers. We observed that senior clinical officers took the discussion of caesarean section indications very serious and this became a standard aspect of the morning handover meeting. With the start of a bachelor training programme for clinical officers in obstetrics and gynaecology, there will be more knowledge and experience on the obstetric level available, hopefully contributing further towards open discussions and thorough decision-making in labour. Alternatively, this could also be an opportunity for a Malawian trained medical officer.

## Conclusions

Our study demonstrates the effect of MDGHTMs on caesarean section rate, as well as on the quality of caesarean section indications. Supervision, stimulating discussion, and audit are major parts of the work of MDGHTMs. Their background training in obstetrics is of benefit. We believe that continuation of training MDGHTMs in order to support health systems in low-income settings is important and more research into their effectivity is helpful.

## Supplementary information

**Additional file 1: Table 1.** Maternal and perinatal outcomes. **Figure 1.** stillbirth and neonatal death rates per quarter.

## Data Availability

The datasets used and/or analysed during the current study are available from the corresponding author on reasonable request.

## Abbreviations

MDGHTM: Medical doctor Global Health and Tropical Medicine
CS: Caesarean section
SB: Stillbirth
NND: Neonatal death
