# Supplementary material for: Impact of Medical Doctors Global Health and Tropical Medicine on decision-making in caesarean section: a pre- and post-implementation study in a rural hospital in Malawi
Source: Hum Resour Health. 2020 Nov 9;18:87. doi: 10.1186/s12960-020-00516-5 (PMC7650186; doi:10.1186/s12960-020-00516-5)
Supplement: Supplementary file 1 — Additional file 1: Table 1. Maternal and perinatal outcomes. Figure 1. stillbirth and neonatal death rates per quarter. [file 12960_2020_516_MOESM1_ESM.docx]

|  | **CS** | | **Other births** | | **Total** | |
| --- | --- | --- | --- | --- | --- | --- |
| **Maternal outcome** | N | % | N | % | N | % |
| Alive | 640 | 99.2 | 2781 | 99.9 | 3421 | 99.8 |
| Maternal death | 4 | 0.6 | 0 | 0.0 | 4 | 0.1 |
| Missing | 1 | 0.2 | 2 | 0.1 | 3 | 0.1 |
| Total | 645 | 100.0 | 2783 | 100.0 | 3428 | 100.0 |
| **Perinatal outcome** |  |  |  |  |  |  |
| Live birth | 643 | 94.3 | 2774 | 98.0 | 3417 | 97.3 |
| Fresh stillbirth | 19 | 2.8 | 17 | 0.6 | 36 | 1.0 |
| Macerated stillbirth | 6 | 0.9 | 17 | 0.6 | 23 | 0.7 |
| Early neonatal death | 7 | 1.0 | 23 | 0.8 | 30 | 0.8 |
| Missing | 7 | 1.0 | 1 | 0.0 | 8 | 0.2 |
| Total | 682 | 100.0 | 2832 | 100.0 | 3514 | 100.0 |

Table 1: Maternal and perinatal outcomes

Figure 1: stillbirth and neonatal death rates per quarter
